# The Effect of Blood Flow Restriction during Low-Load Resistance Training Unit on Knee Flexor Muscle Fatigue in Recreational Athletes: A Randomized Double-Blinded Placebo-Controlled Pilot Study

**DOI:** 10.3390/jcm13185444

**Published:** 2024-09-13

**Authors:** Aleksandra Królikowska, Maciej Daszkiewicz, Julia Kocel, George Mihai Avram, Łukasz Oleksy, Robert Prill, Jarosław Witkowski, Krzysztof Korolczuk, Anna Kołcz, Paweł Reichert

**Affiliations:** 1 Ergonomics and Biomedical Monitoring Laboratory, Department of Physiotherapy, Faculty of Health Sciences, Wroclaw Medical University, 50368 Wroclaw, Poland; maciej.daszkiewicz@student.umw.edu.pl (M.D.); julia.kocel2@gmail.com (J.K.); anna.kolcz@umw.edu.pl (A.K.); 2 Department of Orthopaedic Surgery and Traumatology, Kantonsspital Baselland Bruderholz, 4101 Bruderholz, Switzerland; george.mihai.avram@gmail.com; 3 Orthopaedics and Traumatology Department, Central Military Emergency Hospital Dr. Carol Davila, 010825 Bucharest, Romania; 4 Department of Orthopaedics, Traumatology and Hand Surgery, Faculty of Medicine, Wroclaw Medical University, 50556 Wroclaw, Poland; loleksy@oleksy-fizjoterapia.pl (Ł.O.); jaroslaw.witkowski@umw.edu.pl (J.W.); krzysztof.korolczuk@umw.edu.pl (K.K.); pawel.reichert@umw.edu.pl (P.R.); 5 Department of Physiotherapy, Faculty of Health Sciences, Jagiellonian University Medical College, 31008 Kraków, Poland; 6 Center of Orthopaedics and Traumatology, University Hospital Brandenburg/Havel, Brandenburg Medical School Theodor Fontane, 14770 Brandenburg an der Havel, Germany; robert.prill@mhb-fontane.de; 7 Faculty of Health Sciences Brandenburg, Brandenburg Medical School Theodor Fontane, 14770 Brandenburg an der Havel, Germany

**Keywords:** ischemic training, orthopedics, physical conditioning, physiotherapy, sports injury prevention, sports medicine, vascular occlusion training

## Abstract

**Background/Objectives:** Despite the growing popularity of training with a controlled form of vascular occlusion, known as blood flow restriction (BFR) training, in the rehabilitation of orthopedic patients and sports medicine, there remains ample space for understanding the basis of its mechanism. The pilot study assessed the effect of BFR during a low-load resistance training unit on knee flexor muscle fatigue, intending to decide whether a larger trial is needed and feasible. **Methods**: The study used a prospective, randomized, parallel, double-blind, placebo-controlled design. Fifteen male healthy recreational athletes were randomly assigned to three equal groups: BFR Group, Placebo Group, and Control Group. The primary outcome was the change in the surface electromyography-based (sEMG-based) muscle fatigue index, which was determined by comparing the results obtained before and after the intervention. The intervention was the application of BFR during low-load resistance training for knee flexors. The occurrence of any adverse events was documented. **Results**: In all groups, the sEMG-based fatigue index for semitendinosus and biceps femoris muscles decreased after low-load resistance training, with the largest decrease in the BFR group. Although not statistically significant, BFR showed moderate and large effect sizes for the fatigue index of semitendinosus and biceps femoris, respectively. No adverse events were noted. **Conclusions**: The pilot study suggested that BFR during a low-load resistance training unit might affect knee flexor muscle fatigue, supporting the development of a larger randomized clinical trial.

## 1. Introduction

Blood flow restriction (BFR) training, also known as occlusion or ischemic training, is increasingly capturing interest in sports and rehabilitation. It involves restricting blood flow to the muscles during exercise, typically using bands or devices like specialized tourniquet cuffs, to partially limit arterial blood flow into the working muscles while allowing venous return [1]. BFR training can be considered a technique designed for muscle building that combines metabolic stress generated by vascular occlusion with mechanical tension during exercise [1].

BFR training is most commonly integrated with resistance training, particularly low-load resistance training, which is the focus of the present study [1,2]. This approach has been shown to enhance motor unit recruitment and stimulate muscle hypertrophy by promoting the early activation of fast-twitch (type II) muscle fibers, yielding results comparable to high-load resistance training alone [3,4,5,6].

Apart from application within low-load resistance training, the BFR has also been explored in aerobic and sport-specific exercises [7,8]. The primary reported effects of BFR in this context are increased muscle mass [9] and strength [7,10,11,12], along with improved anaerobic power and aerobic parameters [13,14]. The literature also shows different forms of BFR usage as a rehabilitation modality [3,15,16] or a potential method of muscle mass maintenance in elderly individuals [4,5,17,18].

As recreational athletes often engage in exercise without the supervision of professional trainers and may follow less structured training regimens, investigating the safety and efficacy of BFR during low-load resistance training specifically for this demographic is crucial [17,19,20,21]. Focusing on knee flexor muscles addresses a specific aspect of sports performance and injury prevention [21,22]. Investigations into the impact of BFR during low-load resistance training on muscle local fatigue are topics that can have significant implications for exercise physiology, sports performance, and injury prevention [7,19,23]. BFR training has been proposed to enhance muscle adaptations with low-load resistance exercises [3]. If the BFR during low-load resistance training induces greater fatigue in exercise muscles, it could imply a more efficient and time-effective approach to training for recreational athletes.

Although there is some literature on general muscle fatigue induced by BFR, there is a notable lack of studies specifically addressing local muscle fatigue [24,25,26]. So far, the research on local muscle fatigue has also focused on larger muscle groups, such as the quadriceps, with limited exploration of smaller muscle groups, such as the knee flexors [27]. Additionally, regarding local muscle fatigue, more needs to be understood about how BFR affects individual muscles within these muscle groups, like the semitendinosus and biceps.

Nonetheless, the current literature lacks information about the specific effects of BFR during low-load resistance training on the local fatigue of exercised muscles. One area that has yet to be explored is the impact of BFR on smaller knee muscle groups. Specifically, there is limited research on the effect of BFR on the knee flexors, including individual muscles such as the biceps and semitendinosus. So far, the impact of BFR has mainly been studied in the quadriceps muscle [27]. There already is some evidence regarding the potential adverse effects of BFR training [28,29,30,31]. However, further prospective research is still valuable in establishing its safety profile.

Therefore, the present pilot study aims to explore the effects of BFR during low-load resistance training on knee flexor muscle fatigue in recreational athletes. Specifically, the study seeks to assess the feasibility of the research protocol, refine methodological approaches, and gather preliminary data on the impact of BFR on the knee flexor muscle fatigue index and any associated adverse events. Additionally, this pilot study will inform the practicality of the research protocol, identify potential challenges, and provide effect size estimates for designing adequately powered future studies.

## 2. Materials and Methods

### 2.1. Ethical Considerations

The pilot study was conducted in 2023 in the Ergonomics and Biomedical Monitoring Laboratory, Department of Physiotherapy, Faculty of Health Sciences and Department of Orthopedics, Traumatology and Hand Surgery, Faculty of Medicine at Wroclaw Medical University, Poland. It was carried out following the Declaration of Helsinki and approved by the Bioethics Committee at the Medical University of Wroclaw, Poland (protocol code KB-192/2023, date of approval: 9 March 2023). The participants were recruited between March and April 2023. The study was retrospectively registered at the Australian New Zealand Clinical Trials Registry (ANZCTR): Trial Id: ACTRN12624000488505; registration date 22 April 2024. Before the study, all participants were informed about the aim of the study and the approach to be used and signed an informed consent. The results of the present pilot study are reported according to CONSORT 2010 guidelines [32].

### 2.2. Study Design

The prospective interventional pilot study used a randomized, parallel, double-masked placebo control design. The primary outcome was the change in the surface electromyography-based (sEMG-based) knee flexor muscles fatigue index. The change was determined by comparing the results obtained at baseline (first assessment) and after the intervention (second assessment). The intervention was the application of BFR during a low-load resistance training unit for knee flexors. The intervention was compared against a placebo (inactive intervention) and no intervention. The first assessment was performed at baseline, just before the low-load resistance training unit, and the second assessment was carried out immediately after the training unit. The secondary outcomes include the occurrence of any adverse events. No changes to the study protocol were provided after approval by the local bioethics committee.

### 2.3. Participants

The studied material consisted of the first 15 male volunteers who met all inclusion criteria. The minimal needed sample could not be calculated precisely; the present pilot study aimed to constitute a base for minimal sample calculation for extensive trial purposes. The volunteers were recruited from the students at Wroclaw Medical University, Poland, via advertising on the laboratory website.

Firstly, the volunteers underwent an interview and a physical examination to check the fulfillment of the criteria. The interview had questions regarding age, physical activity level, history of musculoskeletal injuries and diseases in the lower limbs or lower back, presence of pain in the lower limbs or lower back, general health and well-being, and diagnosed systematic disorders. Next, the body mass (kg) and height (cm) were measured, and the Body Mass Index (BMI) was calculated. Consecutively, the physical examination was carried out bilaterally, including inspection and palpation of the knee joint; special tests (Lachman test, anterior drawer test, pivot-shift test, posterior drawer test, valgus and varus stress tests, Apley grinding test, and McMurray test); measurement of knee range of motion using universal goniometer (°); assessment of knee muscles strength using Medical Research Council (MRC) Scale for Muscle Strength (grades 0–5); measurement of knee joint and thigh circumferences using measuring tape (cm) [33,34,35,36]. The dominant lower limb was determined by asking If you would shoot a ball on a target, which leg would you use to shoot the ball? [37].

The inclusion criteria were as follows: male; dominant right lower limb; age 20–30 years; lack of a history of injuries or diseases in lower limbs or lower back; no pain in lower limbs or lower back; no diagnosed systematic illnesses; sports activity on a recreational level; general good health; lack of any contraindications for BFR training, specifically any heart or vessels disorders, venous thromboembolism, antiphospholipid syndrome, family history of venous thromboembolism, thrombophilia, limb infection, diabetes mellitus, cancer, history of vascular transplantation, lymphoedema, or antithrombin deficiency; BMI between 18.50 and 24.99; bilateral full range of knee active motion; bilateral knee muscles strength exceeding grade 5 in MRC; bilaterally negative results of all performed special tests; the between-limb differences in knee joint and thigh circumferences less than 2 cm.

The allocation sequence generation and participant enrollment were performed by researchers who were not involved in applying intervention and outcomes examination. Simple randomization using a randomization table from a statistic book was used. One of the researchers, who was also not involved in applying intervention and outcomes examination, prepared 15 sealed opaque envelopes with the assignment to particular study groups and intervention, placebo, or non-intervention. Therefore, with the use of sealed opaque envelopes, the 15 participants in the study sample were randomly allocated to the three equal studied groups: BFR Group, Placebo Group, and Control Group (allocation ratio 1:1:1). The researcher who applied the intervention was responsible for the assignment to intervention by opening consecutive envelopes. Thus, only the examiner responsible for applying the BFR bands and carrying out the low-load resistance training for knee flexors knew to which group the patient was allocated. The examiner who conducted the sEMG examination and the participants were blinded regarding group allocation. The person conducting the statistical analysis was not aware of which of the groups was the intervention, placebo, or control group.

All the participants visited the laboratory on two separate occasions within one week. On the first occasion, the participants underwent the measurements of the maximal isometric torque of knee flexor muscles. On the second occasion, local muscle fatigue assessment using sEMG was performed just before and immediately after the low-load resistance knee training unit. Depending on the studied group, during the training, the BFR (BFR Group), placebo (Placebo Group), or no intervention (Control Group) was applied. Experienced examiners performed all tests and the training.

On each occasion, the participants wore comfortable sports outfits. Each occasion started with a standardized warm-up on a cycle ergometer. During all measures and training, verbal start and stop commands were used.

### 2.4. Knee Flexor Muscle Maximal Isometric Torque Measurements

The measurements of the maximal isometric torque of knee flexor muscles in the dominant limb separately with the use of an isokinetic dynamometer, namely Biodex System 4 Pro (Biodex Medical Systems, Inc., Shirley, NY, USA), and hand-held dynamometer (K-Force Muscle, Kinvent, Montpellier, France).

Knee flexor muscle strength was measured using an isokinetic dynamometer in a seated position with the examined knee flexed at 90°. The length of the lever arm was 40 cm for all the participants. The trunk was stabilized using belts. The participant’s arms were crossed on his chest, and his head was leaning on the chair. The foot of the examined limb was in a neutral position. Two repetitions of maximal isometric knee flexion were performed, lasting for 5 s and separated by a 30 s break. The higher value was considered the maximal isometric torque (N * m^−1^) for setting the load during resistance training for knee flexor purposes.

The measurement of knee flexor strength using the hand-held dynamometer was carried out supine with the examined limb flexed in hip and knee joints at 90°. The shin of the examined limb was supported on a wooden box. The hand-held dynamometer was placed on the posterior side of the shin at the level of the ankle joint. The participant’s arms were crossed on his chest, and his head was leaning on the floor. The foot of the examined limb was in a neutral position. The participant performed two repetitions of maximal isometric pressure against the hand-held dynamometer that lasted 5 s and were separated by a 30 s break. The higher value was considered the maximal isometric force applied against the device (kg) for setting the load during the muscle local fatigue assessment using sEMG.

### 2.5. Local Muscle Fatigue Assessment

**Figure 1:**
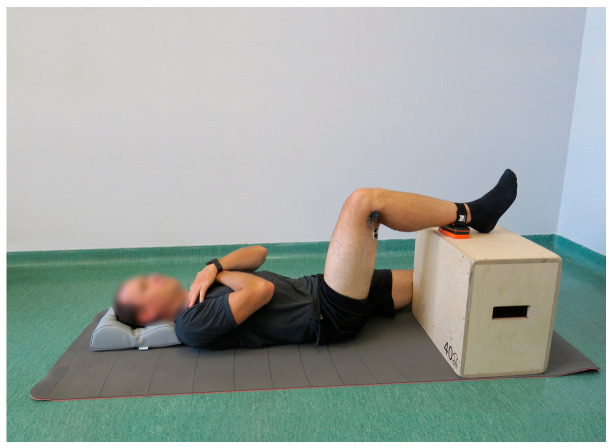
The starting position of the local knee flexors muscles fatigue assessment in the dominant lower limb using surface electromyography, performed during an isometric contraction against a hand-held dynamometer.

Local muscle fatigue assessment using sEMG was performed during a 60 s isometric contraction of the knee flexor muscles against a hand-held dynamometer (K-Force Muscle, Kinvent, Montpellier, France) with a pre-defined resistance constituting 50% of the maximal isometric force applied against the device, measured on a previous occasion. The contraction was controlled with biofeedback. The test was performed in a supine position with the examined limb flexed in hip and knee joints at 90° and the shin of the examined limb supported on a wooden box. The dynamometer was placed on the posterior side of the shin at the level of the ankle joint. The arms of the examined person were crossed on his chest, and his head was leaning on the floor. The foot of the examined limb was in a neutral position. The assessment is presented in Figure 1.

The activity of knee flexor muscles was recorded using a direct transmission system for sEMG, simultaneously using separate channels for semitendinosus and biceps femoris muscles. The signals were registered with a 16-bit accuracy at a sampling rate of 1500 Hz using the Noraxon G2 TeleMyo 2400 unit (Noraxon USA, Inc., Scottsdale, AZ, USA). The sEMG signal was processed using the MyoResearch 3.14.38 software (Noraxon USA, Inc., Scottsdale, AZ, USA). The muscle activity was recorded following SENIAM guidelines; however, the electrodes were placed according to Beretta Piccoli et al. (2014) [38,39]. Before the placement of electrodes, the skin was appropriately prepared, the hair was removed, and the skin was cleaned with alcohol. Disposable dual Ag/AgCl electrodes (Noraxon, Scottsdale, AZ, USA) with low-impedance solid gel, stainless steel snaps, and latex-free, hypoallergenic foam adhesive backing with dimensions 40 × 21 mm and interelectrode distance exceeding 20 mm were used.

**Figure 2:**
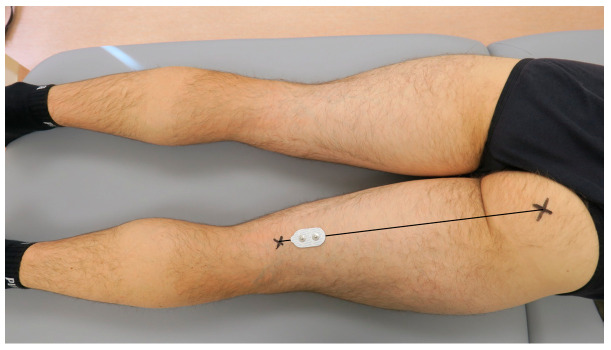
The placement of the surface electromyography dual-electrode for recording the activity of the semitendinosus muscle in the examined dominant lower limb.

**Figure 3:**
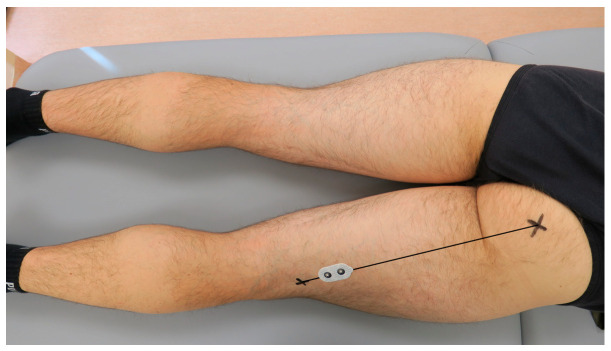
The placement of the surface electromyography dual-electrode for recording the biceps femoris muscle activity in the examined dominant lower limb.

According to Beretta Piccoli et al. (2014), when recording the activity of the semitendinosus muscle, the electrodes should be placed at 0–26% or 74–100% of the length of an imaginary line between the ischial tuberosity and the medial side of the popliteal cavity [39]. For the present study purposes, the dual-electrode was placed at 80% of the length of the mentioned line, as presented in Figure 2. When recording the activity of the biceps femoris muscle, the electrodes should be placed at 0–22% or 72–100% of the length of an imaginary line between the ischial tuberosity and the lateral side of the popliteal cavity. Therefore, for the present study purposes, the dual-electrode was placed at 80% of the length of the mentioned line, presented in Figure 3. Consecutively, the wireless sEMG sensors were fixed to the skin using double-sided tape (Noraxon, Scottsdale, AZ, USA).

The recorded raw sEMG signal was analyzed step-wise in 1 s intervals over 60 s of static-isometric contraction. The mean frequency (Hz) was calculated for each 1 s window. For consecutive analysis purposes, the sEMG-based local fatigue index was calculated separately for the semitendinosus and biceps femoris muscles. It is defined as the ratio of the mean frequency (Hz) in the last second of the isometric contraction to the mean frequency (Hz) in the first second [40].

### 2.6. Occurrence of Adverse Events

A comprehensive approach to monitoring and categorizing adverse events throughout the study was implemented. All participants were observed closely during the study sessions, with particular attention given to any signs of discomfort or unexpected reactions. Immediately after each training session, participants were interviewed in person to capture any immediate adverse events or symptoms they experienced. This face-to-face interaction allowed for detailed reporting and ensured that any adverse events were documented promptly. Additionally, to identify any delayed adverse events, participants were contacted by phone the following day. This follow-up helped in capturing any issues that may not have been immediately apparent.

Adverse events were categorized according to their nature (e.g., discomfort, injury), severity (mild, moderate, severe), duration, and the actions taken (e.g., medical intervention). This categorization enabled examiners to assess the overall safety of the intervention and to determine if any adjustments to the study protocol were necessary.

### 2.7. Blood Flow Restriction

**Figure 4:**
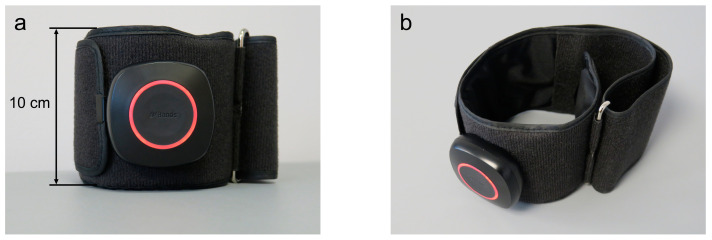
The front view (**a**) and the side view from above (**b**) of the lower limb blood flow restriction cuff that was used for the present study purposes.

In the BFR Group and Placebo Group, a wireless lower limb BFR cuff of a length of 81 cm and a width of 10 cm was used (AirBands, VALD Health, VALD Pty Ltd., Newstead, QLD, Australia) as presented in Figure 4.

**Figure 5:**
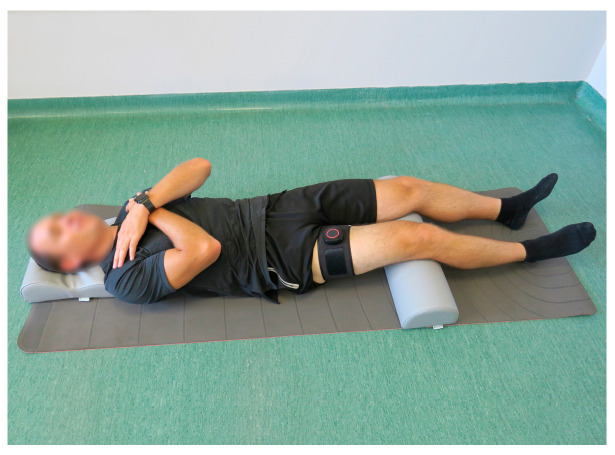
Placement of the blood flow restriction cuff on the thigh of the dominant lower limb at the level of the largest circumference, directly under the inguinal fold.

The cuff had a PVC module attached to a band. The cuff was placed on the thigh of the dominant limb, at the level of the largest circumference, directly under the inguinal fold (Figure 5).

Then, the examiner ensured the module was facing forward and the AirBands logo could be read the right way up. The examiner fastened the cuff in the correct position by wrapping the band through the metal loop, ensuring enough room under the cuff for two fingers between the skin and the band and no residual air in the air bladder. Consecutively, the cuff was paired with the AirBands App installed on the tablet (Galaxy Tab S7 SM-T870, Samsung Electronics Co., Ltd. Suwon-si, Republic of Korea) via Bluetooth.

The degree of BFR was precisely controlled using the AirBands, which were individually calibrated for each participant to determine their unique Limb Occlusion Pressure (LOP)—the minimum pressure required to fully occlude blood flow in the limb. Calibration was conducted with the participant lying supine, with the knee relaxed and supported on a half-moon bolster pillow. During this process, the AirBands cuffs were automatically inflated to identify the maximal limb pressure (100% LOP) before being deflated. For participants in the BFR Group, 80% of their detected LOP was consistently applied throughout the low-load resistance training, in line with the manufacturer’s guidelines. This method ensured a standardized application of 80% LOP across all participants while still tailoring the BFR to each individual’s specific LOP. In the Placebo Group, the cuff remained uninflated during the training sessions.

### 2.8. Low-Load Restriction Training

**Figure 6:**
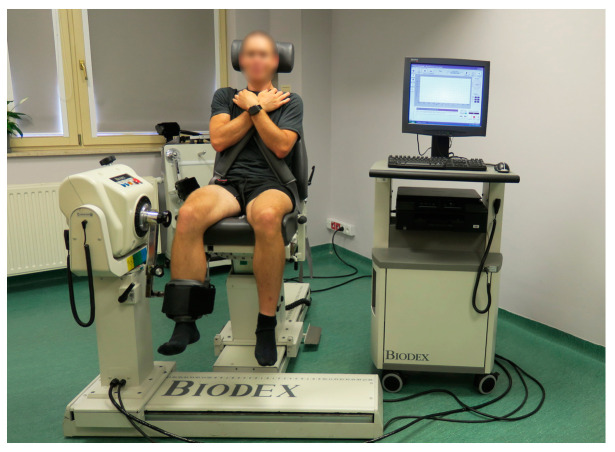
The starting position for the dominant limb low-load resistance training with blood flow restriction for knee flexors using an isokinetic dynamometer.

The unilateral low-load resistance training unit for dominant limb knee flexors was performed using Biodex System 4 Pro (Biodex Medical Systems, Inc., Shirley, NY, USA), as presented in Figure 6. The length of the lever arm was set at 40 cm. The trunk was stabilized using belts. The arms of the participant were crossed on his chest, and his head was leaning on the chair. The foot of the trained limb was in a neutral position. Three consecutive series of alternate concentric repetitions were performed for extension and flexion of the knee joint; however, the resistance was applied only for knee flexion. The pre-defined resistance exceeded 30% of the maximal isometric torque measured on a previous occasion. The four series consecutively comprised 30, 15, 15, and 15 repetitions [3]. Thirty-second-long breaks separated consecutive series.

### 2.9. Statistical Analysis

All statistical analyses were conducted using SPSS Statistics Version 28.0.1.0 (IBM Corp., Armonk, NY, USA) and Microsoft Excel 365 (Microsoft Corp., Redmond, WA, USA). Given the pilot nature of the study, no formal sample size calculation was performed. Descriptive statistics, including arithmetic means and standard deviations (±), were calculated for the studied variables. Statistical significance was set at *p* < 0.05.

The Shapiro–Wilk test was applied to assess normality. Body mass data were normally distributed, so a one-way analysis of variance (ANOVA) was used to compare body mass and height between groups. For age and BMI, which did not meet normality assumptions, the Kruskal–Wallis H test was employed for between-group comparisons.

The primary outcome, the sEMG-based local fatigue index, was analyzed separately for the semitendinosus and biceps femoris muscles. Within-group comparisons of the fatigue index between the first and second assessments were conducted. For the semitendinosus muscle, the Wilcoxon signed-rank test was used for the Placebo and Control groups, while a dependent *t*-test was applied to the BFR group. For the biceps femoris muscle, dependent *t*-tests were used for all groups.

The between-group comparison was based on the change in the index, calculated by subtracting the value obtained during the first assessment from the value obtained during the second assessment. Following the Shapiro–Wilk test, the biceps femoris fatigue index change was compared between the BFR and Placebo groups and between the BFR and Control groups using independent *t*-tests. The change in the semitendinosus fatigue index was compared between the BFR and Placebo groups using the Mann–Whitney U test and between the BFR and Control groups using independent *t*-tests.

Cohen’s d was calculated to measure effect sizes for within-group comparisons (first vs. second assessment) and between-group comparisons of the change in the fatigue index for both the semitendinosus and biceps femoris muscles. Effect sizes were interpreted as small (0.2), medium (0.5), or large (0.8) [41].

## 3. Results

After randomization, no losses or exclusions were carried out. No adverse events were noted. There were no missing data.

Table 1 presents the characteristics of the studied sample. The three studied groups were comparable in terms of age (*p* = 0.402), body mass (*p* = 0.633), body height (*p* = 0.810), and BMI (*p* = 0.386).

Recorded values of the mean frequency of semitendinosus and biceps femoris muscles activity during a 60 s contraction of the examined lower limb, separately for the BFR, Placebo, and Control groups, were included in Supplementary Tables S1 and S2. As raw recorded data have no clinical value, they were not statistically analyzed. They constituted a basis for calculating the sEMG-based fatigue index of the semitendinosus and biceps femoris muscles.

In the BFR, Placebo, and Control groups, the semitendinosus muscle sEMG-based fatigue index was statistically significantly lower during the second assessment when compared to the first assessment, with the *p*-values exceeding *p* = 0.009, *p* = 0.043, and *p* = 0.043, respectively. The details are presented in Table 2.

As Table 2 shows, the changes in the semitendinosus muscle sEMG-based fatigue index between the first and second assessments were the largest in the BFR group compared to the Placebo and Control groups. The changes in the Placebo Group were larger than in the Control Group.

Table 3 shows that changes in the semitendinosus muscle sEMG-based fatigue index between the first and second assessments did not differ statistically significantly between the BFR and Placebo groups (*p* = 0.331). The effect size (Cohen’s d = 0.19) suggests a small magnitude of difference. In contrast, comparing the BFR and Control groups approached statistical significance (*p* = 0.066). The effect size was moderate (Cohen’s d = 0.59), suggesting a more substantial difference between these groups, though it did not reach statistical significance.

In the BFR and Placebo groups, the biceps femoris muscle sEMG-based fatigue index was statistically significantly lower during the second assessment when compared to the first assessment, with the *p*-values exceeding *p* ≤ 0.001 and *p* = 0.018, respectively, as presented in Table 4. In the Control Group, the biceps femoris muscle sEMG-based fatigue index values obtained during the second assessment were smaller than those obtained during the first assessment, but the difference was not statistically significant.

Similarly to the semitendinosus muscle, the changes in the biceps femoris muscle sEMG-based fatigue index between the first and second assessments were the largest in the BFR group compared to the Placebo and Control groups, as presented in Table 4. Also, the changes in the Placebo Group were larger than in the Control Group.

Regarding the biceps femoris muscle sEMG-based fatigue index, the comparison between the BFR and Placebo groups showed a non-significant *p*-value of 0.175, with a moderate effect size of Cohen’s d = 0.52. This suggests that while the effect of BFR relative to Placebo was moderate, it did not reach statistical significance. Conversely, comparing the BFR and Control groups yielded a *p*-value of 0.356 and a large effect size of Cohen’s d = 1.31. Although this difference was not statistically significant, the large effect size indicates a substantial impact of BFR compared to the Control Group.

## 4. Discussion

The present pilot study aimed to explore the effects of BFR during low-load resistance training unit on knee flexor muscle fatigue in recreational athletes. For local muscle fatigue assessment, an sEMG-based fatigue index was used. In all studied groups, the sEMG-based fatigue index of semitendinosus and biceps femoris muscles decreased after the low-load resistance training unit; however, the largest decrease was noted for the BFR Group. Even though the between-group differences were not statistically significant, BFR demonstrated moderate and large effect sizes for the sEMG-based fatigue index of semitendinosus and biceps femoris muscles, respectively. The observed response could constitute a base for formulating the hypothesis for a future prospective randomized clinical trial (RCT) that BFR can induce significant changes in the sEMG-based fatigue index in knee flexor muscles.

Interestingly, the effect size calculation underscored distinct response magnitudes in the two studied muscles. Specifically, the biceps femoris muscle exhibited a more prominent response to low-load resistance training unit than the semitendinosus muscle under BFR conditions compared to the reactions observed in the Control and Placebo groups.

Considering the significance of effect size estimates in instances where *p*-values are non-significant (as shown in Table 3), it is essential to note that the effect size offers insight into the practical, real-world impact of the intervention. Still, in the present pilot study, the non-significant *p*-value indicates that the sample size might have been insufficient to detect the required difference. Consequently, future randomized controlled trials should be designed with a larger sample size to enhance the ability to detect meaningful effects. It is worth adding that the effect size calculations added to the reported *p*-values will inform the sample size estimation of a prospective randomized controlled trial.

Focusing on recreational athletes is important because they represent a significant portion of the population engaged in physical activity. However, they often have limited time for training and may prefer less intensive training methods [42,43]. If BFR proves effective in inducing fatigue during low-load resistance training, it could offer an appealing option for recreational athletes looking to maximize the benefits of their workouts without the need for heavy loads or extended training time.

Understanding the impact of BFR on knee flexor muscle fatigue is particularly relevant for injury prevention and rehabilitation. If BFR can enhance fatigue in these muscles, it may contribute to improved knee muscle strength, potentially reducing the risk of injuries or aiding in rehabilitation post-injury [21,44,45]. The usefulness of BFR combined with low-load resistance training provides the opportunity for rehabilitation under a low-joint-stress environment in postoperative patients [46,47], recreational athletes [5], or the elderly [4], thereby allowing faster postoperative recovery, physical conditioning, and muscle mass maintenance. In postoperative patient cohorts, especially following anterior cruciate ligament reconstruction (ACLR), BFR combined with low-load resistance training has been shown to be beneficial in terms of preserving muscle mass and bone density [46,48]. At the same time, other reports underline the importance of applied physical therapy regimens with intermittent BFR, which do not provide the same results [49,50,51]. Notably, the evidence regarding the effectiveness of BFR therapy in the postoperative period following ACLR predominantly centers on strengthening knee extensor muscles, especially the vastus medialis. There is a noticeable gap in data availability concerning the usage of BFR to increase the strength of knee flexor muscles, even though the weakness of this muscle group constitutes an important issue in the view of graft harvesting and prevention of secondary injuries [21,52,53,54,55,56,57].

Before implementing a novel intervention like BFR on a broader population, assessing its safety and efficacy is crucial [15,58,59,60]. It has been commonly assumed that the risks of BFR training are comparable to those of traditional training [58]. These risks include but are not limited to blood clotting and muscle damage [58,61]. Additionally, research conducted by Królikowska et al. identified that BFR adversely affects joint position sense [62]. Consequently, it is crucial to exercise caution and implement special attention when engaging in BFR training. A pilot study allows for the preliminary investigation of potential adverse effects and the effectiveness of the intervention in a controlled environment. In the present pilot study, no adverse effects were recorded, supporting the safety of establishing an RCT.

The present randomized double-blinded placebo-controlled pilot study is justified to bridge the existing knowledge gap, assess the safety and efficacy of the intervention, optimize study design, estimate sample sizes, minimize bias, and provide relevant information for recreational athletes and the broader field of sports science [63,64,65,66,67,68]. Conducting a pilot study enables researchers to refine the study design and methodology before committing to a larger-scale investigation. This includes assessing the feasibility of recruiting and retaining participants, refining the protocol, and determining appropriate outcome measures. Pilot studies can help estimate the required sample size for a larger trial. Analyzing the data from a smaller group of participants can provide valuable insights into effect sizes, variability, and potential trends that can inform the sample size calculation for a subsequent larger-scale study.

The pilot study, conducted with a sample size of 15, was carried out under ethical, time, personnel, and financial constraints, with the primary goal of providing initial effect size estimates for the intervention and control groups. These effect size estimates, especially when *p*-values are non-significant, offer critical insights into the practical impact of the intervention. The data obtained, specifically the effect size estimates (intervention vs. control, etc.), will be used to calculate the appropriate sample size for a full-scale randomized controlled trial. This future trial will be designed with a larger sample size, informed by these estimates, to improve statistical power and increase the likelihood of detecting the effect of the intervention. Thus, the pilot study results are crucial for informing sample size calculations for the subsequent full trial.

Also, the fundamental role of the Control Group in the present study should not be omitted as it provides a baseline for assessing the natural variability in the sEMG-based fatigue index without any intervention. The observed changes in the Control group offer a crucial reference point, helping to differentiate the effects of the interventions from the normal physiological fluctuations in muscle fatigue. By comparing the Control Group with the BFR and Placebo groups, researchers can more accurately assess the impact of the interventions and understand the extent to which they influence muscle fatigue beyond what would occur naturally.

In this study, participants in the Control Group were not subjected to BFR or any placebo intervention, so their muscle fatigue levels were influenced solely by regular physiological processes and daily activities. The observed changes in the sEMG-based fatigue index within the Control Group, such as the modest reductions in both the semitendinosus and biceps femoris muscles, reflect this natural variability. These changes provide a baseline measure of how muscle fatigue can vary over time due to factors like normal muscle recovery, daily physical activity, and individual differences in muscle endurance. The interventions in the BFR and Placebo groups are expected to induce changes in muscle fatigue beyond what would occur naturally. By comparing the changes in the fatigue index in these groups to those in the Control Group, researchers can isolate the effects of the interventions. This baseline variability helps contextualize the effects observed in the BFR and Placebo groups. For instance, the larger reductions in the fatigue index in the intervention groups suggest that the interventions are effective in influencing muscle fatigue, as these changes exceed the natural variability shown by the Control Group. Understanding this baseline variability allows researchers to identify the true effects of the interventions and assess their significance. Without the Control Group, it would be difficult to determine whether the observed changes in the fatigue index in the intervention groups were due to the interventions themselves or simply a reflection of normal variability.

In terms of limitations, given the BFR influences metabolic pathways that ultimately lead to muscle mass and strength improvement, more extensive follow-up periods must be implemented between baseline assessment, intervention, and a secondary assessment following the intervention. Furthermore, in planning future randomized controlled trials, accounting for the enhanced precision achievable through larger sample sizes is essential, reducing the Type I error rate [64]. Given the detection of substantial effect sizes, the feasibility of conducting a randomized controlled trial appears promising. Therefore, there are no anticipated significant economic burdens on the investigative team, particularly concerning the challenge of recruiting more patients.

Another limitation that needs to be advocated in future trials is that the baseline values of the semitendinosus muscle sEMG-based fatigue index obtained during the first assessment were lower in the Control Group than in the BFR and Placebo groups. We do not know if this could affect the results. Still, in future studies, efforts should be made to obtain comparable baseline values of the fatigue index across the study groups, even though the values are not compared directly between the groups.

Although the blinding methods described in the present pilot study are robust, a few additional steps could be considered to evaluate and confirm the effectiveness of the blinding in future research [69]. One method of ensuring that blinding is effective is to ask the participants and the blinded examiners at the end of the study to guess which group they think the participants were assigned to (BFR, Placebo, or Control). This can help determine if blinding was successful. If their guesses are no better than random chance, it suggests that blinding was effective. The accuracy of the guesses made by participants and examiners regarding group assignments could even be statistically analyzed. Comparing their guesses with the actual allocations can provide a quantifiable measure of the success of blinding. If the accuracy is not significantly better than random chance, this would indicate effective blinding [70]. Also, during the study, whether participants or examiners express any assumptions or suspicions about the group assignments can be regularly assessed. This could be done informally by asking participants or examiners whether they know which group they were assigned to without directly influencing their perception.

Furthermore, future studies should consider the participants’ previous training levels in addition to their amateur classification. The previous training history of participants could significantly influence their response to BFR and resistance training interventions. Individuals with different training backgrounds may have varying levels of muscle conditioning, which could affect the degree of fatigue induced by low-load resistance training with BFR. By stratifying participants based on their training history or including it as a covariate in the analysis, future research can better account for these differences, leading to more precise and generalizable findings.

A notable limitation of the present study is the need for more control or screening for participants’ prior experience with resistance training. This factor could influence the outcomes, as individuals with previous resistance training experience may respond differently to the intervention compared to those without such a background. In future trials, it would be essential to implement a more rigorous screening process to assess and categorize participants’ prior resistance training experience. This could be achieved through detailed questionnaires or interviews that specifically inquire about the type, frequency, and intensity of past resistance training activities. Additionally, stratifying participants based on their training experience or including it as a covariate in the analysis could help control this variable and ensure that the results more accurately reflect the impact of the intervention itself.

The final limitation of this study is the lack of prospective registration, which introduces the potential for retrospective registration bias. Although the study was retrospectively registered with the Australian New Zealand Clinical Trials Registry (ANZCTR) to address this issue, the limitation remains. In the country where the study was conducted, clinical trial registration is primarily focused on studies involving medicinal products, and there is no centralized registry for non-pharmacological interventions like the one presented here. Furthermore, this study was initially a pilot feasibility study intended to inform a larger trial, and at the time, registration was not considered necessary. However, it is now recognized that even pilot studies should be registered to ensure transparency and minimize bias. Importantly, the study protocol has not changed since ethical approval, maintaining consistency in the objectives and methods. Moving forward, the authors are committed to prospectively registering future full-scale trials in international registries to enhance the transparency and credibility of the research.

## 5. Conclusions

The present pilot study suggested that BFR during a low-load resistance training unit might affect knee flexor muscle fatigue in recreational athletes. The study supports the development of a future larger-scale randomized clinical trial on the topic.

## Figures and Tables

**Table 1:** Characteristics of the studied sample.

Studied Group	*n*	Age (Years)	Body Mass (kg)	Body Height (cm)	BMI (kg * m^−2^)
BFR Group	5	23.40 ± 0.55	79.40 ± 9.29	182.80 ± 5.72	23.72 ± 2.05
Placebo Group	5	22.60 ± 0.89	75.20 ± 10.78	179.60 ± 5.68	23.21 ± 2.06
Control Group	5	26.60 ± 2.07	73.20 ± 10.66	180.80 ± 10.85	22.31 ± 1.32
Between-Group *p*-Value		0.402	0.633	0.810	0.386

Values are presented as the arithmetic mean with the corresponding standard deviation (±). BFR, blood flow restriction; BMI, body mass index; *n*, number of participants; *p*, significance level.

**Table 2:** The within-group comparison of the semitendinosus muscle surface electromyography-based index obtained during the first and second assessments in the three studied groups.

Semitendinosus Muscle Surface Electromyography-Based Fatigue Index				
	First Assessment	Second Assessment	Within-Group *p*-Value	Index’s Change
BFR Group	0.95 ± 0.04	0.84 ± 0.08	**0.009**	−0.11 ± 0.05
Placebo Group	0.93 ± 0.03	0.85 ± 0.15	**0.043**	−0.09 ± 0.14
Control Group	0.88 ± 0.14	0.81 ± 0.20	**0.043**	−0.07 ± 0.08

Values are presented as the arithmetic mean with the corresponding standard deviation (±). BFR, blood flow restriction, *p*, significance level. The *p* < 0.05 was indicated in bold. The index’s change refers to the difference in the sEMG-based fatigue index between the first and second assessments.

**Table 3:** Comparison between the BFR Group and Placebo and Control groups in terms of the change in the semitendinosus and biceps femoris muscles surface electromyography-based fatigue index.

Change in the Surface Electromyography-Based Fatigue Index between the First and Second Assessment				
BFR Group				
	Semitendinosus Muscle		Biceps Femoris Muscle	
	Between-Groups *p*-Value	Effect Size (Cohen’s d)	Between-Groups *p*-Value	Effect Size (Cohen’s d)
Placebo Group	0.331	0.19	0.175	0.52
Control Group	0.066	0.59	0.356	1.31

Values are presented as a significance level (*p*) and effect size (Cohen’s d). BFR, blood flow restriction.

**Table 4:** The within-group comparison of the biceps femoris muscle surface electromyography-based fatigue index obtained during the first and second assessments in the three studied groups.

Biceps Femoris Muscle Surface Electromyography-Based Fatigue Index				
	first Assessment	second Assessment	Within-Group *p*-Value	Index’s Change
BFR Group	0.94 ± 0.05	0.83 ± 0.06	**≤0.001**	−0.10 ± 0.02
Placebo Group	0.92 ± 0.09	0.84 ± 0.06	**0.018**	−0.08 ± 0.05
Control Group	0.93 ± 0.06	0.88 ± 0.04	0.067	−0.05 ± 0.05

Values are presented as the arithmetic mean with the corresponding standard deviation (±). BFR, blood flow restriction, *p*, significance level. The *p* < 0.05 was indicated in bold. The index’s change refers to the difference in the sEMG-based fatigue index between the first and second assessments.

## Data Availability

All data generated and analyzed during this study are available as Supplementary Table S3.

## Supplementary Materials

The following supporting information can be downloaded at: https://www.mdpi.com/article/10.3390/jcm13185444/s1, Table S1: Recorded values mean frequency of semitendinosus muscle activity during a 60-s contraction of the examined lower limb. Table S2: Recorded values mean biceps femoris muscle activity frequency during a 60-s contraction of the examined lower limb. Table S3: Raw data generated and analyzed during the present study.
