# Supplementary material for: The Effect of Blood Flow Restriction during Low-Load Resistance Training Unit on Knee Flexor Muscle Fatigue in Recreational Athletes: A Randomized Double-Blinded Placebo-Controlled Pilot Study
Source: J Clin Med. 2024 Sep 13;13(18):5444. doi: 10.3390/jcm13185444 (PMC11432244; doi:10.3390/jcm13185444)
Supplement: Supplementary file 1 [file jcm-13-05444-s001.zip › Supplementary Table S1 .pdf]

**SUPPLEMENTARY TABLE S1** Recorded values mean frequency of semitendinosus muscle activity during a 60-second contraction of the examined lower limb.

| Mean frequency of semitendinosus muscle activity during a 60-second contraction (Hz) | | | | |
|---|---|---|---|---|
| | 1st assessment | | 2nd assessment | |
| | First second | Last second | First second | Last second |
| BFR Group | 125.20 ± 15.29 | 119.00 ± 15.30 | 108.70 ± 11.14 | 91.29 ± 14.68 |
| Placebo Group | 101.33 ± 11.43 | 94.59 ± 11.40 | 126.40 ± 12.26 | 105.74 ± 13.27 |
| Control Group | 106.82 ± 14.69 | 94.28 ± 18.67 | 110.28 ± 18.86 | 87.48 ± 15.59 |

Values are expressed as arithmetic mean and standard deviation (±). BFR, blood flow restriction.
