# Supplementary material for: The Effect of Blood Flow Restriction during Low-Load Resistance Training Unit on Knee Flexor Muscle Fatigue in Recreational Athletes: A Randomized Double-Blinded Placebo-Controlled Pilot Study
Source: J Clin Med. 2024 Sep 13;13(18):5444. doi: 10.3390/jcm13185444 (PMC11432244; doi:10.3390/jcm13185444)
Supplement: Supplementary file 1 [file jcm-13-05444-s001.zip › Supplementary Table S2.pdf]

SUPPLEMENTARY TABLE S2 Recorded values mean biceps femoris muscle activity frequency during a 60-second contraction of the examined lower limb.

Mean frequency of semitendinosus muscle activity during a 60-second contraction (Hz)				
	1st assessment		2nd assessment	
	First second	Last second	First second	Last second
BFR Group	75.96 ± 7.19	71.10 ± 8.17	99.42 ± 15.20	82.46 ± 10.44
Placebo Group	106.56 ± 11.83	98.46 ± 15.81	103.98 ± 6.31	87.62 ± 4.94
Control Group	87.40 ± 14.96	81.52 ± 13.85	76.53 ± 6.80	67.35 ± 3.23

Values are expressed as arithmetic mean and standard deviation (±). BFR, blood flow restriction.
