# Supplementary material for: The Effect of Blood Flow Restriction during Low-Load Resistance Training Unit on Knee Flexor Muscle Fatigue in Recreational Athletes: A Randomized Double-Blinded Placebo-Controlled Pilot Study
Source: J Clin Med. 2024 Sep 13;13(18):5444. doi: 10.3390/jcm13185444 (PMC11432244; doi:10.3390/jcm13185444)
Supplement: Supplementary file 1 [file jcm-13-05444-s001.zip › Supplementary Table S3.pdf]

Table S3. Raw data generated and analyzed during the present study.

Studied group	Mean frequency of biceps femoris muscle activity during a 60-second contraction (Hz) First assessment First second	Mean frequency of biceps femoris muscle activity during a 60-second contraction (Hz) First assessment Last second	Mean frequency of semitendinosus muscle activity during a 60-second contraction (Hz) First assessment First second	Mean frequency of semitendinosus muscle activity during a 60-second contraction (Hz) First assessment Last second	Mean frequency of biceps femoris muscle activity during a 60-second contraction (Hz) Second assessment First second	Mean frequency of biceps femoris muscle activity during a 60-second contraction (Hz) Second assessment Last second	Mean frequency of semitendinosus muscle activity during a 60-second contraction (Hz) Second assessment First second	Mean frequency of semitendinosus muscle activity during a 60-second contraction (Hz) Second assessment Last second
1	79,90	74,20	124,00	110,00	78,70	67,40	93,60	68,40
1	66,90	65,70	121,00	118,00	105,00	93,20	109,00	102,00
1	79,00	73,00	150,00	145,00	112,00	90,80	108,90	94,63
1	84,00	81,90	123,00	116,00	88,40	77,60	125,00	105,00
1	70,00	60,70	108,00	106,00	113,00	83,30	107,00	86,40
2	89,80	88,90	94,60	85,10	98,00	88,80	121,00	102,00
2	114,00	110,00	115,00	108,00	113,00	93,20	128,00	120,00
2	103,00	78,70	100,33	91,85	106,00	79,60	146,00	85,70
2	121,00	118,00	110,00	105,00	97,90	88,40	124,00	115,00
2	105,00	96,70	86,70	83,00	105,00	88,10	113,00	106,00
3	65,90	64,90	86,10	85,40	71,80	64,00	80,40	73,50
3	108,00	101,00	112,00	111,00	71,10	66,30	114,00	104,00
3	85,60	77,40	126,00	109,00	76,53	67,35	119,00	102,00
3	89,60	89,00	109,00	99,80	75,20	66,40	107,00	97,80
3	87,90	75,30	101,00	66,20	88,00	72,70	131,00	60,10
